# Bartter Syndrome With Normal Aldosterone Level and Acute Pancreatitis: An Unusual Presentation

**DOI:** 10.1002/ccr3.73418

**Published:** 2026-09-01

**Authors:** Noor Ayaz, Matti Ullah, Waqar Ahmad, Farhan Ullah, Ikram Ullah Khan, Fahad Naim, Awais Naeem, Fatima Sajjad, Kamil Ahmad Kamil

**Affiliations:** ^1^ Department of Medicine Khyber Teaching Hospital Peshawar Pakistan; ^2^ Department of Medicine Khyber Medical College Peshawar Pakistan; ^3^ Internal Medicine Department Nasrat Bashir Curative Hospital Kandahar Afghanistan

**Keywords:** acute pancreatitis, Bartter syndrome, hypokalaemia, metabolic alkalosis, normo‐aldosteronism

## Abstract

Bartter syndrome is a rare autosomal recessive renal tubulopathy, which classically presents with hypokalemic metabolic alkalosis, hyperreninemia, and hyperaldosteronism. We report an atypical presentation in a 16‐year‐old male who was diagnosed following an initial manifestation of acute pancreatitis. Key biochemical findings included profound hypokalemia (1.4 mEq/L), metabolic alkalosis, hyperreninemia (218.5 uIU/mL), and hypercalciuria, yet with a normal serum aldosterone level, which is a notable deviation from the classic biochemical profile. The acute pancreatitis was attributed to severe hypokalemia‐induced pancreatic ductal dysfunction. This case underscores that a normal aldosterone level does not exclude Bartter syndrome and emphasizes the importance of considering this possibility in patients with hypokalemic alkalosis and renal salt‐wasting. Furthermore, it highlights severe hypokalemia as a potential, life‐threatening precipitant of acute pancreatitis, warranting prompt investigation in affected patients presenting with acute abdominal pain.

**Key Clinical Message**

Severe hypokalemia in Bartter syndrome can precipitate acute pancreatitis, a life‐threatening complication that should be considered in patients presenting with acute abdominal pain. Furthermore, a normal aldosterone level does not exclude the diagnosis, as normo‐aldosteronism is a recognized variant.

## 1 Introduction

Bartter syndrome is a rare autosomal recessive renal tubulopathy (estimated incidence of 1.2 per million) resulting from defective salt reabsorption in the thick ascending limb of the loop of Henle [1]. The impaired function of the Na + ˗K + ˗2Cl‐ cotransporter (NKCC2) leads to a classic triad of hypokalemic metabolic alkalosis, salt‐wasting, and hyperreninemic normotension [2]. Clinical presentation; however, can be heterogeneous. Notably, normal levels of aldosterone are a rare but documented finding that challenges diagnostic norms [1, 2]. Furthermore, while renal manifestations dominate, severe extra‐renal complications occur. Acute pancreatitis, attributed to hypokalaemia‐induced pancreatic ductal dysfunction, represents one such life‐threatening yet reported phenomenon.

We present a case of Bartter syndrome in a 16‐year‐old male, remarkable for its atypical presentation with acute pancreatitis and a normal aldosterone level, underscoring the diagnostic challenges and expanded clinical spectrum of this disorder.

## 2 Case History

A 16‐year‐old South Asian male presented to the emergency department with a 7‐day history of severe, continuous abdominal pain radiating to his back. The patient was notably underweight with a body weight of 34 kg and a frail body habitus.

On physical examination, the patient appeared ill‐looking and exhibited signs of dehydration. Vital signs were as follows: blood pressure 100/70 mmHg, pulse 68 beats per minute, oxygen saturation 97% on room air, with no postural drop. He had characteristic “Bartter facies,” including a triangular face, pouting mouth, and protruding ears. The rest of the systemic examination, including cardiovascular, respiratory, and neurological systems, was unremarkable. Abdominal examination revealed a soft, non‐tender, and non‐distended abdomen with a notable absence of bowel sounds on auscultation.

### 2.1 Investigations and Differential Diagnosis

Initial laboratory investigations confirmed acute pancreatitis, with serum amylase and lipase elevated to three times the upper limit of normal as described below in Table 1. An abdominal ultrasound demonstrated a bulky pancreas consistent with inflammation along with no cholelithiasis, biliary sludge, or biliary ductal dilation, effectively ruling out gallstone pancreatitis. CT scan of abdomen with pancreatic protocol showed a slightly bulky and irregular pancreas with no remarkable peripancreatic soft tissue stranding or collection, findings consistent with acute pancreatitis CTSI 2.

**TABLE 1:** Key diagnostic laboratory findings at presentation.

Parameter	Patient value	Normal range
Serum potassium (K+)	1.4 mEq/L	3.5–5.0 mEq/L
Serum bicarbonate (HCO3‐)	31.6 mEq/L	22–28 mEq/L
Arterial pH	7.445	7.35–7.45
pCO2	47.1 mmHg	32–45 mmHg
Base excess	+7.5 mmol/L	˗2 to +2
Plasma renin activity	218.5 uIU/mL	2.8–39.9 uIU/mL Supine position 4.4–46.1 uIU/mL Upright position
Serum aldosterone	3.5 ng/dL	1.76–23.2 Supine position 2.52–39.2 Upright position
24‐h urine calcium	316 mg/day	< 150 mg/day for low to average diet
Serum magnesium	2.41 mg/dL	1.5–2.5 mg/dL
Spot urine sodium	40 mmol/L	Variable
Spot urine potassium	15 mmol/L	Variable
Spot urine chloride	46 mmol/L	> 20 mmol/L suggests renal wasting
Urine osmolality	67 mOsm/kg	500–800 mOsm/kg
Urine K/Cr ratio	> 15 mEq/g approx. 65	Renal potassium wasting
Serum lipase	800 U/L	< 45 U/L
Serum amylase	759 U/L	< 90 U/L
Serum calcium	9.5 mg/dL	8.5–10.5 mg/dL
Procalcitonin	< 0.1 ng/mL	< 0.1 ng/mL
Triglycerides	80 mg/dL	< 150 mg/dL
Cholesterol	110 mg/dL	< 200 mg/dL

A thorough evaluation for common causes of acute pancreatitis was undertaken to exclude alternative causes. The patient and family explicitly denied any alcohol consumption and there was no history of abdominal trauma. He was on no chronic medications and had no recent drug exposures that could trigger pancreatitis. Serum triglycerides (80 mg/dL) and calcium (9.5 mg/dL) were within normal limits. Procalcitonin was < 0.1 ng/mL, making an infectious etiology unlikely. Autoimmune markers including ANA were also negative. After systematically excluding these common causes, severe hypokalemia remained the most plausible etiologic factor for acute pancreatitis in this unique clinical context.

Baseline laboratory findings were significant for profound hypokalemia (K+ 1.4 mEq/L) and metabolic alkalosis (HCO3–31.6 mEq/L). Other results are summarized in Table 1.

The patient's past medical history was significant for polydipsia and polyuria since childhood. The family history was notable for the consanguineous marriage of his parents but was negative for renal or endocrine disorders. He had no known drug allergies and was on no other chronic medications.

**FIGURE 1:**
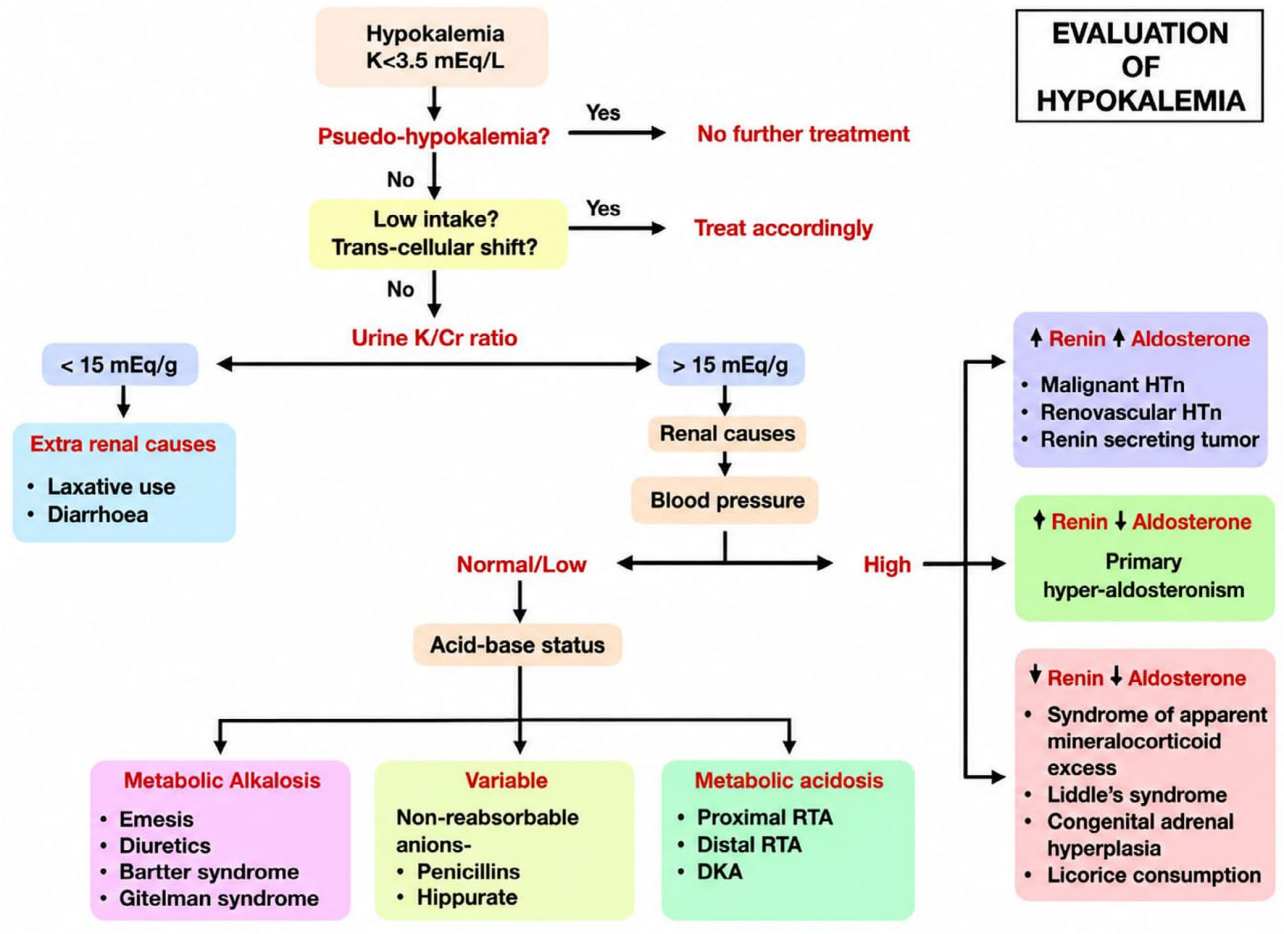
Flow chart for evaluation of hypokalemia.

A structured hypokalaemia workup was initiated based on Figure 1. Pseudohypokalaemia was excluded by immediate sample processing. Inadequate dietary intake was ruled out by dietary history. A high urinary potassium‐to‐creatinine ratio (65 mEq/g; normal < 15 mEq/g) confirmed renal potassium wasting. Spot urine electrolytes revealed sodium 40 mmol/L, potassium 15 mmol/L, and chloride 46 mmol/L. The elevated urine chloride (> 20 mmol/L) confirmed renal salt‐wasting and effectively excluded surreptitious vomiting as a cause.

The presence of normotension and metabolic alkalosis narrowed the differential diagnosis to inherited tubulopathies. Surreptitious diuretic use was excluded through: (1) explicit denial by patient and family, (2) prolonged inpatient observation over 10 days, during which the patient remained under supervision including a supervised 24‐h urine collection, with no evidence of diuretic administration. The finding of hypercalciuria with a normal serum magnesium level (2.41 mg/dL) was pathognomonic for Bartter syndrome over Gitelman syndrome.

Arterial blood gas analysis revealed: pH 7.445 (reference range 7.35–7.45), pCO2 47.1 mmHg (32–45 mmHg), and calculated bicarbonate 31.6 mmol/L. Base excess was +7.5 mmol/L (reference range ˗2–+2). These findings confirmed primary metabolic alkalosis with appropriate respiratory compensation (expected pCO2 for this degree of metabolic alkalosis is approximately 45 mmHg), ruling out acidosis or mixed acid–base disorders.

The diagnosis was further supported by marked hyperreninemia and, interestingly, a normal aldosterone level. Hormonal assays were performed on a blood sample drawn around 11:30 AM with the patient in the supine position (lying in bed). Plasma renin concentration was measured by chemiluminescence immunoassay (Chughtai Lab) and was markedly elevated at 218.5 uIU/mL (reference range for supine position: 2.8–39.9 uIU/mL). Serum aldosterone was 3.56 ng/dL (reference range for supine position: 1.76–23.2 ng/dL), which is within the normal range despite significant hyperreninemia—a recognized but uncommon finding in Bartter syndrome.

### 2.2 Outcomes and Follow‐Up

The patient's acute pancreatitis was managed supportively with IV fluids and oral analgesia. For the definitive management of Bartter syndrome, he was started on potassium supplements. He was prescribed a tablet potassium chloride (NEO K) one tablet three times daily. For gastroprotection, a proton‐pump inhibitor was prescribed, and tablet Paracetamol (Panadol) was provided as needed. The hormonal profile of the patient was also obtained. He was at Tanner Stage 4, and his LH, FSH, GH, and testosterone levels were normal.

The patient's condition improved steadily. The abdominal pain resolved, and his electrolyte levels were stabilized. He was discharged in a stable condition with a plan for long‐term follow‐up in the Endocrinology clinic. The patient and his father provided written informed consent for publication of this case report and expressed their gratitude for the care provided.

## 3 Discussion

Bartter syndrome is a well‐characterized autosomal recessive renal tubulopathy, classically defined by hypokalemic metabolic alkalosis, hyperreninemia, and hyperaldosteronism resulting from defective salt reabsorption in the thick ascending limb of the loop of Henle [3]. The case we present is remarkable because it deviates from this classic presentation in two significant ways: the presence of a normal serum aldosterone level (normo‐aldosteronism) and the initial manifestation of the disease as acute pancreatitis. This combination of atypical biochemical and clinical features provides a unique opportunity to discuss the diagnostic challenges and expanded clinical spectrum of this rare disorder.

The finding of a normal serum aldosterone level in our patient, despite significant hyperreninemia (218.5 uIU/mL), is a key atypical feature. In classic Bartter syndrome pathophysiology, the chronic volume depletion and impaired sodium chloride delivery to the macula densa lead to intense activation of the renin‐angiotensin‐aldosterone system (RAAS) [4]. However, normo‐aldosteronism, while uncommon, is a recognized phenomenon. This presentation is most frequently associated with Bartter syndrome Type 3, which results from mutations in the CLCNKB gene encoding the chloride channel CIC‐Kb [5]. The proposed mechanisms for this dissociation include a blunted adrenal response to angiotensin II or, more notably, a direct suppressive effect of chronic, severe hypokalaemia on aldosterone synthesis itself [6]. Our case underscores that the diagnosis of Bartter syndrome should not be dismissed based on a normal aldosterone level alone. Instead, clinicians must rely on the full clinical picture, including the hallmark findings of renal salt‐wasting, hypokalaemia, metabolic alkalosis, and hypercalciuria.

Furthermore, our patient's initial presentation with acute pancreatitis represents a potentially novel and severe extra‐renal complication. While there is some association of chronic pancreatitis with Bartter syndrome, acute pancreatitis is not a well‐documented feature of Bartter syndrome in the medical literature. A comprehensive search did not yield any confirmed case reports of this specific association, making our case particularly instructive. The pathogenesis in our patient is directly linked to his profound hypokalaemia (K+ 1.4 mEq/L). Pancreatic bicarbonate secretion is critically dependent on the function of basolateral potassium channels (e.g., BK channels) to maintain the electrical driving force for apical chloride and bicarbonate secretion [7]. Severe hypokalemia impairs K+ efflux, dissipating this gradient and leading to a failure of fluid secretion. The resulting viscous pancreatic juice is prone to forming obstructing protein plugs, leading to ductal hypertension, premature intracellular enzyme activation, and ultimately, autodigestion and acute pancreatitis [8]. This process may be potentiated by the elevated levels of prostaglandin E2 that are characteristic of the Bartter syndrome state, which can promote and sustain local inflammatory pathways [4]. A thorough workup excluded other common causes of pancreatitis, making hypokalaemia‐induced pancreatic dysfunction the most plausible mechanism in this unique presentation.

When placed in the context of the existing literature, the uniqueness of our case becomes evident. To the best of our knowledge, this is one of the first detailed reports of an adolescent patient with Bartter syndrome presenting with acute pancreatitis coinciding with normo‐aldosteronism. While isolated normo‐aldosteronism in Bartter syndrome has been previously documented [1, 2], its co‐occurrence with acute pancreatitis as the presenting feature is exceptionally rare. This simultaneous occurrence of two rare features suggests a potentially distinct and severe clinical phenotype.

The primary limitation of our report is the lack of genetic confirmation, which is often constrained by cost and accessibility in our clinical setting. Nevertheless, the diagnosis remains robust based on the clinical and biochemical criteria fulfilled. The lessons from this case are immediately applicable to clinical practice.

## 4 Conclusion

This case provides two critical insights for clinicians. First, the diagnostic criteria for Bartter syndrome should be applied comprehensively, and normo‐aldosteronism should not deter clinicians from making the diagnosis when other features are compelling. Second, severe hypokalaemia and metabolic alkalosis in Bartter syndrome should be recognized as a potential precipitant of acute pancreatitis. We recommend that any patient with Bartter syndrome presenting with acute abdominal pain should undergo prompt measurement of serum amylase and lipase to rule out this serious complication. Increased vigilance can lead to earlier intervention and improved patient outcomes.

## Author Contributions

**Noor Ayaz:** data curation, investigation, writing – original draft. **Matti Ullah:** data curation, visualization, writing – original draft. **Waqar Ahmad:** methodology, resources, validation. **Farhan Ullah:** resources, validation, writing – original draft. **Ikram Ullah Khan:** resources, validation, visualization. **Fahad Naim:** project administration, supervision, writing – review and editing. **Awais Naeem:** project administration, supervision, writing – review and editing. **Fatima Sajjad:** resources, visualization. **Kamil Ahmad Kamil:** supervision, writing – review and editing.

## Funding

The authors have nothing to report.

## Consent

The authors certify that they have obtained all appropriate written patient consent forms. In the form, the patient has given his consent for his images and other clinical information to be reported in the journal. The patient understands that his name and initials will not be published, and due efforts will be made to conceal his identity.

## Conflicts of Interest

The authors declare no conflicts of interest.

## Data Availability

The data that support the findings of this study are available on request from the corresponding author. The data are not publicly available due to privacy or ethical restrictions.
